# Advances in Spatial Multi-Omics in Gastric Cancer

**DOI:** 10.3390/cells15060535

**Published:** 2026-03-17

**Authors:** Hongfei Yan, Yang Liu

**Affiliations:** 1 Department of Pathology, Yale School of Medicine, New Haven, CT 06510, USA; 2 Department of Neurology, Yale School of Medicine, New Haven, CT 06510, USA; 3 Yale Cancer Center, Yale School of Medicine, New Haven, CT 06510, USA

**Keywords:** gastric cancer, spatial multi-omics, tumor microenvironment, therapeutic resistance, precision medicine

## Abstract

Gastric cancer (GC) remains a major global health burden, with its unfavorable prognosis primarily driven by extensive tumor heterogeneity. Traditional bulk omics, while informative, are inherently limited by the averaging effect of diverse cell populations and fail to capture the critical spatial molecular disparities within the tumor and its microenvironment (TME). Single-cell omics can capture cellular heterogeneity but lack spatial context. Therefore, there is an urgent clinical need for spatial multi-omics to provide a high-definition dissection of GC heterogeneity and to optimize therapeutic efficacy. This review first outlines briefly the evolution of spatial technologies, including transcriptomics, proteomics, metabolomics, genomics and epigenomics, and their transformative applications in GC research. We further explore how these platforms refine molecular classification beyond traditional models, identify next-generation biomarkers, and decode the intricate cellular interactions governing immune evasion and metastasis. Next, we highlight the pivotal role of spatial profiling in unravelling the multidimensional mechanisms of resistance to chemotherapy, targeted therapy and immunotherapy. Finally, we address current technical bottlenecks and discuss prospects for clinical translation.

## 1. Introduction

Gastric cancer (GC) is a major global health burden with high morbidity and mortality [1,2]. Despite advances in multimodal treatment strategies, including surgery, chemoradiotherapy and immunotherapy, the 5-year survival rate for GC remains below 40% [3]. Current GC diagnosis relies on imaging studies combined with endoscopic biopsy, which not only enables initial tumor cell characterization but also detects key molecular markers such as HER2, microsatellite stability and Claudin 18.2 [4]. These results support multidisciplinary teams in formulating treatment plans that are more personalized than previous approaches, while surgical specimens further allow pathologists to analyze the tumor’s molecular spectrum to inform subsequent therapy personalization. The poor clinical outcome of GC is largely attributable to the extensive heterogeneity of the tumor. This heterogeneity exists not only between patients (inter-patient heterogeneity) but also within distinct regions of the same tumor (intra-tumoral heterogeneity) and across the tumor microenvironment [5,6]. It serves as a fundamental driver of poor therapeutic response, recurrence, and drug resistance, posing a significant barrier to the development and implementation of precision oncology [7,8,9]. An in-depth characterization of GC from a spatiotemporal perspective, encompassing molecular, cellular and tissue levels, is essential for deciphering tumor heterogeneity, optimizing therapeutic efficacy and ultimately improving patient survival.

Although traditional bulk omics offer a broad overview of molecular landscapes, they are intrinsically constrained by signal averaging across heterogeneous cell populations, thereby obscuring intra-tumoral heterogeneity. To address this, single-cell and spatial multi-omics have emerged, enabling researchers to achieve precise localization and deep characterization of genes, proteins and metabolites at unprecedented resolution while preserving spatial coordinates [10,11,12]. These emerging technologies enable high-resolution characterization of the tumor microenvironment, distinguishing immune “hot” and “cold” regions and revealing how spatial organization contributes to immune evasion [13,14]. By tracing clonal trajectories and identifying spatially distinct drug-resistant subpopulations, spatial omics provides direct evidence for the “seed-and-soil” hypothesis and local resistance mechanisms [11,15]. Moreover, spatial technologies have proven to be useful for enhancing early diagnosis by pinpointing molecular abnormalities in precancerous lesions [16,17]. By adding a new spatial dimension to our understanding of tumor progression and therapy response, spatial omics is steering gastric cancer research toward a more sophisticated stage of precision medicine.

Acknowledging the transformative potential of spatial omics and the clinical demand for understanding GC heterogeneity, this review provides a systematic overview of the latest progress. We briefly summarize the principles and evolution of spatial multi-omics, then highlight their roles in gastric cancer initiation, progression, and clinical management, with emphasis on insights into tumor biology. We also discuss current technical and translational challenges. This review aims to provide forward-looking guidance for advancing precision diagnosis and treatment in GC.

## 2. Spatial Omics Technologies

### 2.1. Spatial Transcriptomics

**Figure 1:**
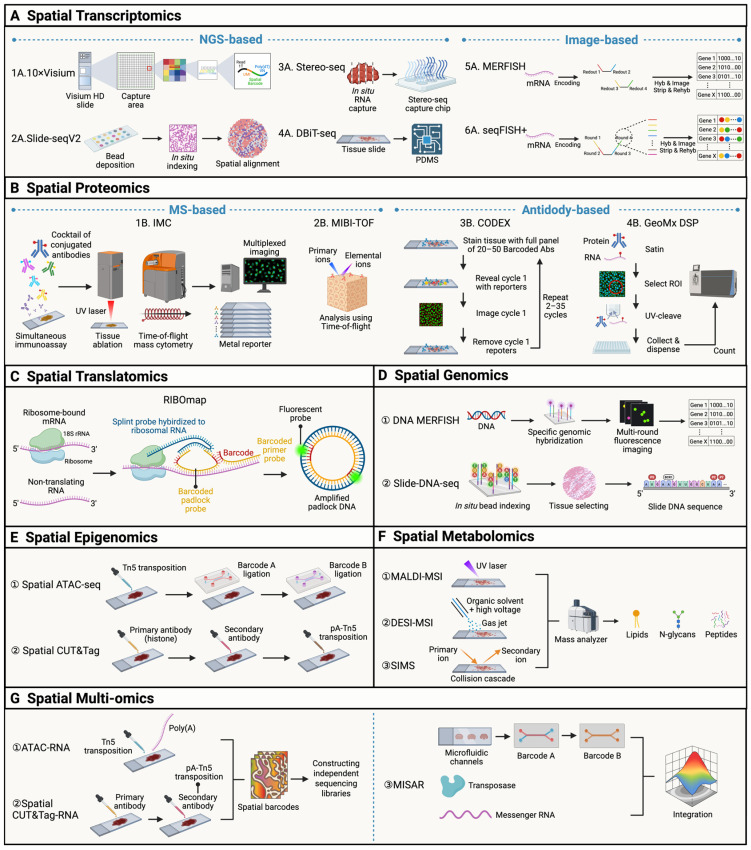
Overview of spatial omics technologies across molecular modalities. (**A**) Spatial transcriptomics methods, including NGS-based approaches (e.g., Visium, Slide-seqV2, Stereo-seq, DBiT-seq) and image-based technologies (e.g., MERFISH, seqFISH), enabling spatially resolved RNA profiling through barcoded capture or multiplexed imaging. (**B**) Spatial proteomics platforms comprising mass spectrometry-based methods (e.g., IMC, MIBI-TOF) and antibody-based approaches (e.g., CODEX, GeoMx DSP) for highly multiplexed protein detection in tissue sections. (**C**) Spatial translatomics, exemplified by RIBOmap, capturing ribosome-associated mRNA to map translational activity in situ. (**D**) Spatial genomics technologies such as DNA MERFISH and Slide-DNA-seq for mapping DNA copy number and genomic loci within intact tissues. (**E**) Spatial epigenomics approaches including spatial ATAC-seq and spatial CUT&Tag for profiling chromatin accessibility and histone modifications with spatial resolution. (**F**) Spatial metabolomics methods (e.g., MALDI-MSI, DESI-MSI, SIMS) enabling in situ detection of metabolites, lipids, glycans, and peptides. (**G**) Spatial multi-omics strategies integrating multiple molecular layers—such as ATAC-RNA, CUT&Tag-RNA, and microfluidic barcoding platforms (e.g., MISAR)—to reconstruct spatially resolved regulatory and molecular interactions. Created in https://BioRender.com (accessed on 2 February 2026).

Spatial transcriptomics (ST) is a cornerstone of spatial omics, mapping gene expression while preserving tissue architecture [18]. It comprises two main technical frameworks, next-generation sequencing (NGS)-based and image-based approaches. NGS-based platforms, including 10 x Visium [19], Slide-seqV1/V2 [20,21], Stereo-seq [22], DBiT-seq [23] and patho-DBiT [24], utilize spatially barcoded probes to capture Mrna [19,25]; 10 x Visium and Visium HD provide regional expression profiles (55 μm or 2 μm resolution), while Slide-seq achieves near-single-cell resolution (10 μm) with enhanced sensitivity [10]. Stereo-seq further reaches subcellular resolution (0.5 μm) via DNA nanoball arrays, enabling large-scale transcriptomic mapping. DBiT-seq (Deterministic Barcoding in Tissue for spatial omics sequencing) uses microfluidic channel-guided DNA barcodes applied in two perpendicular flows to generate unique 2D pixel barcodes directly on tissue sections, achieving ~10 μm spatial resolution. It enables spatial profiling of transcripts and proteins via DNA-conjugated antibodies [23]. Patho-DBiT is an optimized version for clinical FFPE samples (including archived tissues), allowing simultaneous detection of mRNA, non-coding RNA, splice isoforms, and genome-wide SNVs to resolve malignant subclones. Although capable of cellular-level spatial mapping, it does not achieve true single-cell resolution and relies on computational tools such as iStar for refinement [24]. Image-based platforms (MERFISH and seqFISH+) rely on cyclic in situ hybridization (ISH). MERFISH [26] achieves subcellular RNA localization through combinatorial encoding, while seqFISH+ utilizes sequential hybridization to detect thousands of genes [27,28,29]. Although offering superior resolution, these methods involve complex workflows and intensive data analysis. By integrating molecular profiles with spatial context, ST has become indispensable for deconstructing tumor heterogeneity, mapping cellular interactions within the TME and identifying spatial biomarkers associated with cancer progression and metastasis [30,31] (Figure 1A, Table 1).

### 2.2. Spatial Proteomics

Spatial proteomics focuses on the distribution and quantification of proteins, categorized into mass spectrometry (MS)-based and antibody-based platforms [10]. MS-based technologies, notably Imaging Mass Cytometry (IMC) and Multiplexed Ion Beam Imaging (MIBI-TOF), utilize metal-tagged antibodies for high-resolution detection [32,33]. IMC couples laser ablation with mass spectrometry to achieve multi-channel imaging at subcellular resolution; while it preserves tissue architecture, it is limited by high costs and low throughput [34,35]. MIBI-TOF employs ion beam scanning on FFPE samples, offering superior multiplexing capabilities, though data standardization remains a challenge [36,37]. Antibody-based technologies include CODEX [38] and GeoMx DSP [39]. CODEX utilizes DNA-barcoded antibodies and iterative hybridization cycles to detect over 20 proteins simultaneously, ensuring high spatial integrity but requiring long experimental durations [38]. GeoMx DSP uses photocleavable indexing for flexible Region of Interest (ROI) selection, enabling simultaneous RNA and protein profiling in FFPE samples; however, it lacks single-cell resolution and offers relatively lower throughput [39]. Collectively, spatial proteomics provides a critical foundation for characterizing protein expression heterogeneity within the tumor and TME (Figure 1B, Table 1).

### 2.3. Spatial Translatomics

Spatial translatomics directly maps protein translational activity within a spatial context [10]. Represented by RIBOmap, this technology utilizes specialized probe libraries to target ribosome-bound mRNA (actively translating mRNA) in situ [40,41]. These probes, which contain ribosome-binding motifs, spatial barcodes, and sequencing adapters, are amplified and sequenced in situ to enable quantitative assessment of translational activity. This approach overcomes the loss of spatial context inherent in traditional translatomics, enabling researchers to correlate transcript abundance with actual protein synthesis efficiency. Although currently constrained by limited throughput and a focus on key functional genes, spatial translatomics is a powerful tool in oncology. It reveals real-time protein synthesis regulation and identifies selective translation events triggered by microenvironmental stressors, such as hypoxia or nutrient deprivation (Figure 1C, Table 1).

### 2.4. Spatial Genomics

Spatial genomics integrates genomic profiling with spatial positioning to resolve the distribution of structural variants, copy number variations (CNVs) and clonal evolution, overcoming the loss of spatial context in traditional genomics [42]. Key technologies include DNA MERFISH26 and Slide-DNA-seq [43]. DNA MERFISH utilizes a multi-round in situ hybridization strategy with encoding probes targeting specific genomic loci, such as oncogene amplification regions or translocation breakpoints [26]. It provides subcellular resolution of genomic alterations across tissue sections. In contrast, Slide-DNA-seq employs bead-array technology to transfer DNA fragments onto spatially barcoded beads. By linking genomic sequences with spatial coordinates, it is better suited for analyzing genomic heterogeneity across large-scale tissues [43]. By directly coupling genetic aberrations with spatial locations, spatial genomics avoids the “averaging effect” of bulk sequencing. It enables the mapping of clonal expansion trajectories, the localization of driver mutations or chromosomal rearrangements, and the correlation of genomic instability with microenvironmental features or metastatic sites. This provides critical histological evidence for understanding tumor pathogenesis and metastatic dynamics (Figure 1D, Table 1).

### 2.5. Spatial Epigenomics

Spatial epigenomics resolves the spatial heterogeneity of epigenetic regulation, primarily through Spatial ATAC-seq [44] and Spatial CUT&Tag [45]. Both technologies rely on Tn5 transposase-mediated in situ labeling and spatial barcoding [45,46,47]. In Spatial ATAC-seq, fixed and permeabilized tissue sections are treated with Tn5 transposase complexes that specifically insert into and label open chromatin regions. Subsequently, a microfluidic chip delivers orthogonal spatial barcodes to index each location. Following tissue lysis and library construction, bioinformatics is used to reconstruct the spatial map of chromatin accessibility [44]. Spatial CUT&Tag employs specific primary antibodies to target histone marks, such as H3K4me3 (active promoters), H3K27ac (active enhancers) and H3K27me3 (repressive regions). Secondary antibodies then recruit pA-Tn5 transposase for site-specific labeling and spatial barcoding. This method yields high-resolution nucleosome positioning with minimal non-specific background noise (Figure 1E, Table 1) [45].

### 2.6. Spatial Metabolomics

Spatial metabolomics utilizes mass spectrometry imaging (MSI) to map the in situ distribution and concentration gradients of metabolites, such as lipids, amino acids and carbohydrates. The field is dominated by three mass spectrometry imaging technologies: matrix-assisted laser desorption/ionization mass spectrometry imaging (MALDI-MSI), desorption electrospray ionization–mass spectrometry imaging (DESI-MSI), and secondary ion mass spectrometry (SIMS). MALDI-MSI employs a laser to excite a matrix-coated sample, achieving resolutions of 10–50 μm (reaching 4 μm with advanced systems) [48]. It offers high sensitivity for both large and small molecules but requires matrix application and a vacuum environment. DESI-MSI uses a charged solvent spray to simultaneously desorb and ionize analytes [49]. It is matrix-free and operates under ambient pressure, making it ideal for rapid clinical analysis of fresh or frozen tissues, though its resolution is generally lower (50–200 μm). Secondary ion SIMS utilizes high-energy ion beams to achieve subcellular resolution (<1 μm) [50]. However, it is restricted to small molecules/elements and requires conductive coatings and an ultra-high vacuum. Spatial metabolomics excels at revealing metabolic reprogramming, such as metabolic disparities between the tumor core and margin, and dissecting nutrient competition between malignant and immune cells [51,52]. These insights provide direct evidence for identifying metabolic targets and developing metabolic inhibitors (Figure 1F, Table 1).

### 2.7. Spatial Multi-Omics

Spatial multi-omics technologies integrate two or more omics layers within the same spatial context, providing a holistic view of molecular complexity and regulatory networks in tissues. By avoiding batch effects from separate sample analyses, these technologies enable precise correlation of molecular events across different regulatory layers, which is crucial for decoding complex biological processes and disease mechanisms (Figure 1G, Table 1).

#### 2.7.1. Transcriptomics and Epigenomics

The technology focuses on co-profiling transcriptional activity and epigenetic regulation to reveal how spatial variations in chromatin states drive gene expression. Spatial ATAC-RNA-seq [53] co-profiles chromatin accessibility and gene expression in the same tissue section, leveraging Tn5 transposase-mediated labeling of open chromatin and in situ capture of poly(A)-tailed mRNA, followed by spatial barcoding to index both datasets to the same coordinates. Its workflow includes treating fixed sections with Tn5 transposase, capturing mRNA with poly(T) probes, introducing spatial barcodes and constructing separate libraries for sequencing, with bioinformatics integration uncovering regulatory relationships like enhancer–promoter interactions, valuable for studying tumor heterogeneity and development [53]. Spatial CUT&Tag-RNA-seq [53] simultaneously profiles histone modifications and RNA expression, using specific primary antibodies against histone marks, secondary antibodies recruiting pA-Tn5 transposase for site-specific labeling, and spatial barcoding to index both signals, yielding high-resolution nucleosome positioning alongside gene expression data to clarify how histone modifications spatially regulate transcriptional programs [53]. Recently, spatial DNA methylation and transcription detection [54] technology was also reported using spatial barcoding technology. MISAR-seq, a microfluidic-based technology, integrates spatial barcoding with transposase-mediated epigenetic labeling and mRNA capture, enabling the identification of key transcription factors and gene regulatory networks, particularly during corticogenesis [55].

#### 2.7.2. Transcriptomics and Proteomics

Technologies combined transcriptomics and proteomics enabled in situ spatial correlation between RNA and protein levels. Core technologies synchronously capture both molecular signals via ADTs and targeted probes, followed by spatial barcoding for localization. Spatial-CITE-seq [56] retains whole-transcriptome coverage, facilitating immune cell subset characterization and RNA biomarker validation in the tumor microenvironment. Stereo-CITE-seq leverages DNA nanoball arrays to achieve precise correlation across large tissue areas at a subcellular resolution of 0.5 μm [57]. GeoMx DSP is compatible with FFPE samples, supporting region-specific ROI selection and targeted co-detection [39]. SPOTS and SM-Omics use Visium-like oligo(dT)-incubated arrays for signal capture, with SM-Omics offering high-throughput and automated capabilities for large-scale profiling [58,59]. STARmap PLUS combines antibody staining and chemical labeling, achieving a high resolution of 200–300 nm and suitability for brain tissues and disease models such as Alzheimer’s disease, enabling spatial correspondence between gene expression and protein biomarkers [60]. MOSAICA targets RNA and protein with primary hybridization probes and double-ended secondary probes, using lifetime imaging and combinatorial encoding with error-correction cycles to achieve multi-omics co-mapping in situ, suitable for complex tissue samples [61].

## 3. Spatial Multi-Omics Approach in GC

GC often presents with insidious onset and non-specific symptoms, leading to late-stage diagnosis for most patients and the loss of opportunities for curative treatment [4]. Furthermore, the profound heterogeneity of GC frequently results in drug resistance and poor therapeutic outcomes. Clinical oncologists are currently focused on identifying distinct molecular subtypes and developing novel diagnostic and therapeutic biomarkers to achieve precision medicine for GC. Spatial multi-omics technologies have demonstrated promising potential in the field of GC.

### 3.1. Advancing Molecular Classification in GC

The current mainstream molecular classifications of GC are primarily defined by The Cancer Genome Atlas (TCGA) and the Asian Cancer Research Group (ACRG). The TCGA classification categorizes GC into four subtypes: EBV-positive (EBV+), characterized by EBV infection, frequent PI3K-AKT mutations and DNA hypermethylation; Microsatellite Instability (MSI), defined by a high mutational burden and mismatch repair defects; Chromosomal Instability (CIN), the most prevalent subtype featuring significant CNVs and TP53 mutations; and Genomically Stable (GS), exhibiting low mutation rates and frequent CDH1 or RHOA mutations [62]. Similarly, the ACRG classification identifies four subtypes: MSI, consistent with TCGA’s MSI and associated with the best prognosis; MSS/EMT, characterized by high mesenchymal marker expression, high metastatic risk and the worst prognosis; MSS/TP53- (inactive), with the highest TP53 mutation frequency; and MSS/TP53+ (active), often associated with EBV infection [63]. Other notable classifications include the Singapore classification (proliferative, metabolic and mesenchymal subtypes) [64] and various Chinese cohorts [62,65]. However, without exception, these classifications rely on bulk tissue analysis, which fails to capture intra-tumoral molecular disparities.

Numerous studies have leveraged spatial metabolomics and MALDI-MSI to construct GC classifications based on metabolic features. Hao Chen et al. identified three metabolic signature clusters (MSCs): MSC1 (upregulated TCA cycle/lipid metabolism; TP53/RHOA mutations) correlates with favorable prognosis and immunotherapy response; MSC2 (elevated nucleotide/amino acid metabolism; intestinal-type/MSI-enriched) shows intermediate prognosis; and MSC3 (enhanced carbohydrate/energy metabolism; diffuse-type/CDH1 mutations) exhibits the worst prognosis and poor immunotherapy efficacy. Spatial metabolomics confirmed significant disparities in the distribution of metabolites, such as arachidonic acid in MSC1, histidine/uracil in MSC2 and cholesterol sulfate in MSC3, revealing metabolic architectures distinct from normal mucosa [66]. For HER2-positive advanced GC, Jun Wang et al. utilized MALDI-IMS and K-means clustering to identify nine metabolic subregions. High metabolic heterogeneity (high Simpson Index) was linked to Trastuzumab sensitivity and better survival, with 82% of responders exhibiting high heterogeneity compared to 44% in the resistant group [67]. Another study proposed three tumor-specific (T1-T3) and three stroma-specific (S1-S3) subtypes; the T1 subtype (active nucleotide metabolism; MSI-H/EBV+) showed the best prognosis and an 82% Trastuzumab sensitivity rate, while the T2 subtype (immune-cold; carbohydrate/amino acid metabolism) had only 44% sensitivity [68]. Characterizing the immune microenvironment is another key strategy.

Xiongyan Wu et al. used the Visium platform and scRNA-seq to identify seven spatial meta-programs (MPs). While MP3 and MP4 relate to CD8^+^ T cell cytotoxicity and exhaustion, MP7 represents a highly malignant state where cells secrete GDF15 to recruit and transform fibroblasts into myCAFs via TGFBR2; these myCAFs then secrete RSPO3 to activate EGR1, promoting the MP7 state and establishing an immunosuppressive niche [69]. Joel Veas Rodriguez et al. combined WES and spatial proteomics to categorize gastric adenocarcinomas into Inflammatory (52%) and Non-Inflammatory (48%) types. The Inflammatory type features high immunogenicity but significant T cell exhaustion (high PD-1/CTLA-4), while the Non-Inflammatory type shows T cell exclusion, VEGFA overexpression and worse outcomes [70]. Regarding chemoresistance, GC was classified into three platinum-resistance subtypes (CS1-CS3). CS2 (stroma-enriched; ECM-activated) exhibited the worst prognosis and lowest drug sensitivity. Spatial transcriptomics revealed that highly resistant malignant cells cluster locally, surrounded by stromal cells to form an “immune-privileged” niche [71]. Finally, for poorly cohesive carcinoma (PCC), Hung-Hsuan Yen et al. used GeoMx DSP to resolve molecular differences between PCC-NOS and SRCC. PCC-NOS was associated with larger tumor volumes, advanced N-stage, and poorer 1-year PFS (39.5% vs. 75.0%). Notably, MMP7 was identified as a key marker in PCC-NOS, correlating with perineural invasion and early disease progression [72].

Overall, novel spatial molecular subtypes maintain clear consistency with traditional TCGA/ACRG classifications. In terms of consistency, spatial subtypes align with the core stratification logic of traditional classifications: for example, MSI-enriched spatial metabolic subtypes (e.g., MSC2) correspond to TCGA’s MSI subtype, Inflammatory immune subtypes overlap with TCGA’s EBV+/MSI subtypes, and Non-Inflammatory subtypes are consistent with TCGA’s CIN/GS subtypes, ensuring continuity with the established GC molecular classification system. However, spatial classification offers distinct spatial advantages. First, it breaks the “averaging effect” of bulk tissue analysis in traditional classifications, capturing intra-tumoral spatial heterogeneity that TCGA/ACRG cannot resolve, and refining a single traditional subtype into multiple spatially distinct subgroups with heterogeneous prognosis and treatment responses. Second, it integrates tumor–stroma interaction context, directly reflecting the spatial crosstalk between malignant cells and the microenvironment—a dimension ignored by traditional classifications that focus solely on tumor cell intrinsic characteristics. Third, it has direct clinical translational value, linking molecular subtypes to therapeutic response to provide precise treatment guidance, whereas traditional classifications primarily serve prognostic stratification. Fourth, it achieves high-resolution subtype delineation, identifying focal, spatially restricted subtypes undetectable by bulk-based traditional classifications, enabling targeted intervention for key pathogenic regions.

Despite the emerging role of spatial omics in developing GC molecular classifications, current research is largely limited to the integration of a single spatial technology with traditional bulk omics. This lack of synergy across multiple spatial layers, such as transcriptomics, metabolomics and proteomics, hinders a comprehensive capture of multidimensional spatial heterogeneity. Furthermore, spatial genomics and translatomics remain underutilized, potentially leading to the loss of critical genomic-level insights in GC stratification. To deeply resolve GC heterogeneity, it is essential to establish refined sub-classifications for different pathological and molecular subtypes based on integrated spatial data. Addressing these technological gaps and cross-modal integration challenges will be the primary focus for future spatial omics-driven GC molecular research.

### 3.2. Discovery of Novel Biomarkers and Therapeutic Targets in GC

**Figure 2:**
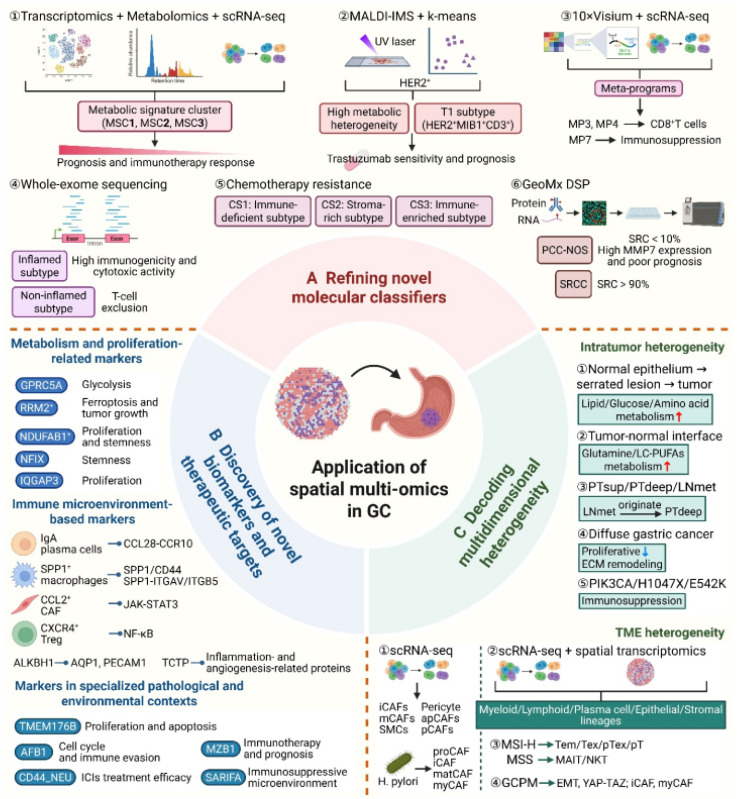
Applications of spatial multi-omics in gastric cancer (GC). This schematic illustrates how integrated spatial transcriptomics, metabolomics, genomics, and proteomics refine molecular classification, identify therapeutic biomarkers, and decode tumor heterogeneity in GC. (**A**) Multimodal approaches define novel metabolic and immune subtypes. (**B**) Spatial profiling further uncovers metabolism- and immune-related biomarkers and (**C**) resolves intra-tumoral and tumor microenvironment heterogeneity across epithelial, stromal, and immune compartments, informing prognosis and therapeutic targeting. Created in https://BioRender.com (accessed on 2 February 2026).

The identification of GC biomarkers remains a research focal point for precision medicine. Current clinical biomarkers are primarily categorized into diagnostic and therapeutic types. Diagnostic markers, such as CEA and CA125, offer convenience but suffer from poor specificity due to interference from benign gastrointestinal diseases, failing to meet the requirements for early precision diagnosis [73,74]. Therapeutic markers, including HER2, Claudin 18.2, FGFR2 and PD-L1, serve as critical criteria for patient stratification in targeted or immunotherapy [4]. However, given the profound histological and molecular heterogeneity of GC, existing biomarkers provide limited coverage for individualized therapy. Consequently, oncologists are committed to discovering novel candidates. Yet, most current candidates derive from bulk sequencing studies, which masks molecular disparities driven by intra-tumoral heterogeneity (ITH) and limits their clinical translational value. Spatial omics technologies address this by providing a “spatiotemporal” dimension, offering a robust framework for the discovery and development of next-generation precision biomarkers (Figure 2, Table 2).

#### 3.2.1. Metabolism and Proliferation-Related Markers

ST has identified several biomarkers linked to metabolic and proliferative activities in GC. GPRC5A, a glycolysis-related gene specifically enriched in the tumor core, correlates with enhanced glycolytic activity and serves as an early diagnostic marker [75]. RRM2+ hyper-proliferative cells cluster spatially within tumor tissues and regulate ferroptosis via the p53 pathway; their inhibitor, osalmid, demonstrates significant anti-tumor potential [76]. At the tumor–stroma interface, the NDUFAB1+ tumor subtype has been identified as a driver of progression due to its high cell cycle activity and stemness, regulated by the transcription factor ELK4 [77]. Furthermore, ST precisely distinguished eight functional clusters in GC tissues, including cancerous, stromal, necrotic, vascular and smooth muscle areas, and identified a “Cancer1” subpopulation with high expression of stem cell markers, subsequently revealing NFIX as a key molecule regulating cancer stem cell-like properties [78]. IQGAP3, acting as a hub for KRAS and TGF-β signaling, maintaining the balance between proliferative (Ki67-high) and slow-cycling (Ki67-low) functional niches, and driving malignant progression [79] (Figure 2B).

#### 3.2.2. Immune Microenvironment-Based Markers

Spatial omics has revolutionized the discovery of immune biomarkers by clarifying cell-to-cell dependencies. IgA^+^ plasma cells spatially neighbored GC cells and interacted via the CCL28-CCR10 axis; while targeting CCL28 alone was insufficient, its combination with anti-PD-L1 therapy significantly enhanced anti-tumor efficacy [80]. SPP1^+^ macrophages represent a critical M2-type subset enriched in deep tumor tissues [81]. They co-localized with epithelial cells to activate pro-tumorigenic pathways via the SPP1/CD44 axis, indicating a poor prognosis [93]. These macrophages also correlated positively with GREM1^+^ CAFs, with which they formed an immunosuppressive niche through collagen signaling and the SPP1-ITGAV/ITGB5 axis [94]. C-C motif chemokine ligand 2 (CCL2), a pivotal member of the CC chemokine family primarily secreted by fibroblasts and macrophages, mediates the specific chemotaxis and infiltration of immune cells via its receptor CCR2, playing a crucial role in immune recruitment within the TME [82,83]. Utilizing Visium spatial transcriptomics, Sung Hak Lee et al. identified the spatial co-localization of CCL2^+^ CAFs and STAT3-activated macrophages. The former recruited myeloid cells and activated the JAK-STAT3 pathway through CCL2 secretion, establishing an immunosuppressive niche. Neutralization or knockdown of CCL2 inhibited myeloid cell migration and STAT3 phosphorylation, thereby reducing CD8^+^ T cell exclusion and suppressing tumor growth [84]. Other spatial markers include CXCR4, which is enriched in Tregs within oxidative stress zones and linked to immunotherapy resistance [85], and ALKBH1, which co-localized with macrophage infiltration as an independent prognostic indicator [86]. Additionally, high plasma levels of TCTP—which spatially co-localized with tumor cells to limit T cell infiltration—were associated with poor outcomes in patients receiving chemo-immunotherapy [87] (Figure 2B).

#### 3.2.3. Markers in Specialized Pathological and Environmental Contexts

Spatial profiling has provided unique insights into specific GC cohorts. In young gastric cancer (GCY), TMEM176B was identified as a novel target regulating proliferation and apoptosis, while spatial data intuitively mapped the high histological heterogeneity between the tumor core region (TCR) and cancer-adjacent tissues (CATs) that TCR are predominantly composed of epithelial cells, whereas the CATs contain a mixture of epithelial cells, B cells, and fibroblasts, providing a visual representation of the histological heterogeneity in GCY [88]. Environmental factors like aflatoxin B1 (AFB1) were found to drive immune evasion via the MAPK3–FOXM1–Cyclin E axis [89]. Within the inflammatory niche, CD44^+^ neutrophils (CD44_NEU) correlated with low immunotherapy response and decreased significantly following immune checkpoint inhibitor (ICI) treatment, serving as a potential predictive marker [90]. In studies of tertiary lymphoid structures (TLSs), mTLS-positive TME are characterized by abundant infiltration of CD3^+^ T cells, CD8^+^ T cells and M1-type macrophages. A plasma cell-based MZB1 score (MPS) was developed: high MPS scores predicted better responses to neoadjuvant anti-PD-1 therapy and lower recurrence risk [91]. Stroma AReactive Invasion Front Area (SARIFA), characterized by direct contact between tumor cells and adipocytes, is a poor prognostic factor in GC, with SARIFA-positive patients exhibiting significantly shortened overall survival [92]. The analysis of ST showed that in SARIFA-positive tumors, high expression of FABP4 in macrophages and reduced pro-inflammatory cytokines at the invasive front indicate a specialized immunosuppressive microenvironment and poor survival, providing a novel prognostic biomarker for GC [95] (Figure 2B).

While spatial omics has identified numerous biomarkers associated with GC prognosis and therapy, several critical limitations remain. First, functional validation is insufficient. Most studies rely on spatial localization and correlation analyses to infer biomarker functions, lacking rigorous in vivo and in vitro experiments to delineate their regulatory mechanisms. Furthermore, the discovery of most biomarkers has not fully leveraged the “spatial” advantages of these technologies or adequately accounted for the ITH of GC, which remains a primary obstacle to their clinical translation.

## 4. Decoding Multidimensional Heterogeneity in GC

Characterizing the heterogeneity of GC represents a pivotal application of spatial omics. The profound heterogeneity of GC is the primary driver of therapeutic resistance, recurrence, metastasis and divergent prognoses [7,8]. Therefore, the precise deconvolution of these heterogeneous features is essential in overcoming current clinical bottlenecks. While traditional transcriptomic technologies reveal differential gene expression, they lack critical information regarding spatial localization, failing to reconstruct intercellular interactions and functional zoning within the TME. The advent of spatial omics has enabled the precise coupling of gene expression profiles with spatial coordinates, providing a revolutionary tool to decode GC heterogeneity from a “spatial dimension”. This section focuses on the significant applications of spatial omics in GC research, organized into two dimensions, the clonal diversity of intra-tumoral malignant cells and the cellular compositional heterogeneity of the TME. By elucidating the core value of this technology, we can uncover the molecular mechanisms of GC heterogeneity and guide the identification of precision therapeutic targets.

### 4.1. Intratumor Heterogeneity

Utilizing spatial multi-omics technologies, researchers have mapped the molecular distribution across distinct regions of GC tissues. By tracing the stepwise metabolic reprogramming from normal epithelium to serrated lesions and finally to tumor, studies have revealed a progressive upregulation of sulfated lipids and enhanced glucose phosphorylation. While oxidative phosphorylation pathway genes are highly expressed in serrated lesions, tumor tissues exhibit enhanced synthesis of arginine, proline and polyamines. Concurrently, an “immune-enriched interface cluster” at the tumor–normal boundary (comprising plasma cells and follicular B cells) displays activated glutamine metabolism and elevated long-chain polyunsaturated fatty acids, uncovering a space-specific metabolic competition mechanism between immune and malignant cells [96]. Employing DSP on 64 GC patients, Raghav Sundar et al. identified significant gene expression disparities between the superficial primary tumor (PTsup), deep primary tumor (PTdeep) and matched lymph node metastases (LNmet). Specifically, 43% of genes were differentially expressed between PTsup and PTdeep, and 38% between PTsup and LNmet, whereas only 16% differed between PTdeep and LNmet. Notably, next-generation sequencing (NGS) showed that 40% of mutations were exclusive to PTdeep and/or LNmet, while only 6% were found in PTsup. Progressive changes in CNVs from PTsup toward PTdeep/LNmet suggest that LNmet more likely originates from the deep tumor regions, providing a molecular basis for tracing the origins of GC metastasis [97]. Diffuse Gastric Cancer (DGC), characterized by diffuse infiltration of malignant cells, typical signet ring cell (SRC) morphology, and with its signet-cell-predominant variant (a major subtype) recognized as the most anaplastic and difficult to treat—featuring a tendency for early metastasis due to poor cell cohesion—is a highly aggressive subtype with poor prognosis due to late diagnosis and frequent peritoneal metastasis [98,99]. In early-stage SRC lesions, the transcriptome resembles normal gastric epithelium but shows reduced CDH1 expression, enrichment of extracellular matrix (ECM) remodeling genes and a proliferative indolent phenotype. Conversely, advanced stages exhibit TP53 and ERBB3 mutations, recovery of CDH1 expression and upregulation of pro-proliferative genes (MYC, MET), cell cycle regulators and Wnt signaling [100]. The Wnt signaling pathway is a master regulator of DGC differentiation; its activity is significantly higher in poorly differentiated cells (PDCs) and invasive cells compared to differentiated SRCs. This undifferentiated state and invasive capacity are maintained via a dual mechanism: early-stage ECM remodeling (interaction between Collagen I deposition and integrinα2β1) and advanced-stage autocrine secretion of Wnt ligands (WNT3A, WNT6) and SFRP2 [101]. Furthermore, Jie Chai et al. utilized DESI-MSI spatial metabolomics to segment gastric signet ring cell carcinoma (GSRC) into normal, poorly differentiated and GSRC regions. They found that lipids such as Phosphatidylethanolamine N-methyl-phosphatidylethanolamine (PE-NMe), Phosphatidylethanolamine (PE), and Sphingomyelin (SM), etc. were significantly upregulated in GSRC but downregulated in poorly differentiated adenocarcinoma. Glycerophospholipid metabolism, glycerolipid metabolism, and fat digestion/absorption were identified as the core dysregulated lipid pathways in GSRC, providing deep metabolic insights into DGC [102]. Finally, heterogeneity within PIK3CA driver mutations significantly impacts the immune microenvironment and therapeutic response. The H1047X mutational subtype exhibits higher Microsatellite Instability (MSI-H) and better response to ICIs, with significant elevation of VISTA and GZMB in immune zones, whereas the E542K mutational subtype shows the lowest PD-L1 positivity [103].

### 4.2. TME Heterogeneity

Spatial omics have enabled the precise identification and mapping of diverse immune cell subtypes and their distributions in GC. CAFs, a core stromal component of GC, exhibit significant subclonal differentiation and spatial heterogeneity. By integrating scRNA-seq and ST, Xijie Zhang et al. characterized six CAFs subtypes: inflammatory (iCAFs), pericytes, matrix (mCAFs), antigen-presenting (apCAFs), smooth muscle cells (SMCs) and proliferative (pCAFs). Notably, pericytes served as a critical origin for iCAFs, mCAFs and apCAFs, while apCAFs localized in close proximity to malignant cells, facilitating space-specific communication via distinct ligand–receptor pairs [104]. In *H. pylori*-associated GC, CAFs were classified into proCAF, iCAF, matCAF and myCAF subtypes. *H. pylori* infection induced high expression of THBS1 and ZFP36, which fostered an immunosuppressive niche by recruiting Tregs and inhibiting cytotoxic T cell activation, respectively [105]. Furthermore, the integration of scRNA-seq and ST identified 34 unique cell lineages in GC, categorized into five major clusters, i.e., myeloid, lymphoid, plasma, epithelial and stromal; DGC was characterized by a significantly higher proportion of plasma cells [106]. Further investigation revealed that B cells in DGC were more enriched within tumor nests than in the intra-tumoral stroma; DGC patients with low CD20 expression faced a poorer prognosis, though adjuvant chemotherapy may improve their survival [107]. Regarding molecular subtypes, MSI-H GC showed significantly elevated proportions of effector memory T cells (Tem), exhausted T cells (Tex), proliferative exhausted T cells (pTex) and proliferative T cells, with persistent activation of effector markers and IFN-γ signaling. Conversely, microsatellite-stable (MSS) GC was more enriched with mucosal-associated invariant T (MAIT) and natural killer T (NKT) cells [108]. Spatial omics also provide significant advantages in exploring the heterogeneity of GC metastasis. For gastric cancer peritoneal metastasis (GCPM), Raghav Sundar’s team integrated WES, WTS and NanoString GeoMx DSP to identify unique molecular signatures, including EMT, YAP-TAZ pathway activation and enrichment of iCAFs and myCAFs—features distinct from liver metastases. Peritoneal tissues could be classified into PMN-early (benign-quiescent) and PMN-late (inflammatory) types; the latter was associated with poor prognosis and suggested that a pre-metastatic niche (PMN) exists even during early cancer stages [109]. TLSs in GCPM also exhibit marked heterogeneity. mTLSs were enriched in B cell and MHC-II antigen-presentation pathways, with increased CD8^+^ memory T cells and plasma cells, whereas iTLS correlated with T cell exhaustion and M2 macrophage activation. iTLS were more prevalent in peritoneal metastases, contributing to a more pronounced immunosuppressive profile [110]. Finally, although gastric cancer brain metastasis (GCMB) occurs in less than 1% of cases, it carries an extremely poor prognosis [111]. Research indicated that brain metastases utilized two co-existing blood supply strategies: angiogenesis and blood vessel co-option. Tumors with a high EMT stated tend to favor blood vessel co-option and exhibited higher immunoreactivity. In contrast, angiogenic regions were primarily located at the tumor periphery, enriched in hypoxia pathways and M2 macrophages, creating an immunosuppressive environment that explained the limited efficacy of anti-angiogenic therapies [112].

## 5. Unraveling the Mechanisms of Therapeutic Resistance in GC

**Figure 3:**
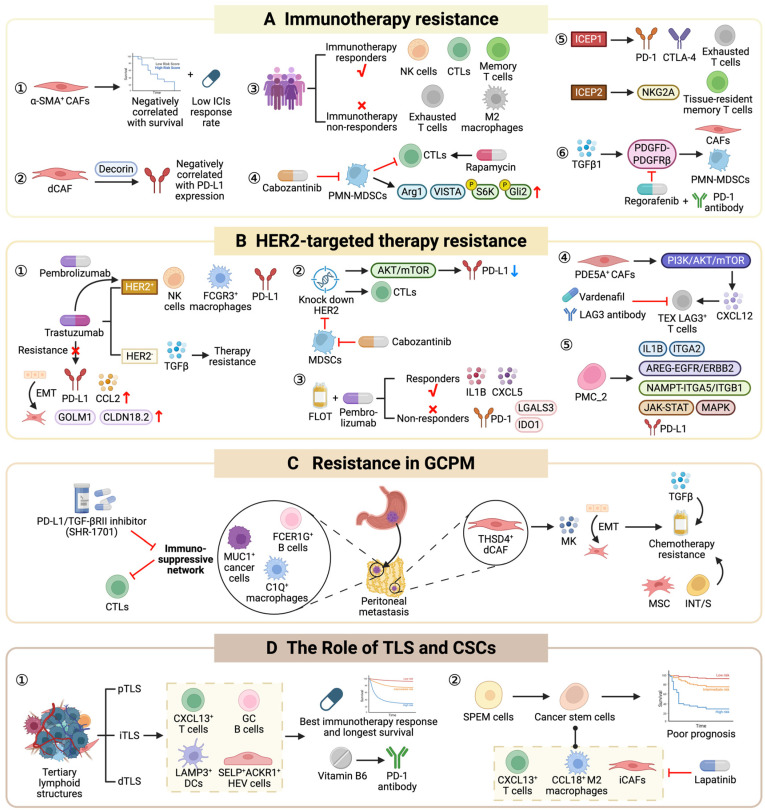
Mechanisms of therapeutic resistance and immune modulation in gastric cancer. This schematic summarizes molecular and cellular mechanisms underlying resistance to (**A**) immunotherapy and (**B**) HER2-targeted therapy, resistance in (**C**) gastric cancer peritoneal metastasis (GCPM), and the (**D**) roles of tertiary lymphoid structures (TLSs) and cancer stem cells (CSCs). Created in https://BioRender.com (accessed on 2 February 2026).

The clinical management of GC has evolved into a comprehensive multimodal diagnostic and therapeutic system centered on surgical intervention. While radical resection offers a favorable prognosis for early-stage patients, the majority of cases are diagnosed at advanced stages, requiring systemic strategies such as chemotherapy, targeted therapy, immunotherapy and combination regimens [4,113]. Chemotherapy, utilizing agents like fluoropyrimidines and platinum, remains the foundational treatment to control disease progression [113,114]. Targeted therapies focusing on specific markers—such as HER2, Claudin18.2 and FGFR2—have substantially extended the survival of patients harboring specific drivers [115,116,117,118]. Meanwhile, immunotherapy has emerged as a transformative hope for late-stage patients, particularly demonstrating robust clinical efficacy in the MSI-H subtype or those with a combined positive score (CPS) ≥ 5 [119,120,121]. Despite these therapeutic milestones, resistance remains the core challenge leading to treatment failure, recurrence and metastasis. This intractable problem is fundamentally driven by the profound heterogeneity of GC. Traditional molecular techniques lack the resolution to precisely capture the spatial evolution of malignant molecular signatures, the regional disparities in drug target expression, or the dynamic, localized interactions between tumor, immune and stromal cells. In this context, the advent of spatial multi-omics provides a pivotal framework to break through these clinical bottlenecks, offering critical insights for developing strategies to precisely reverse therapeutic resistance (Figure 3).

### 5.1. Immunotherapy Resistance

Spatial profiling has fundamentally refined our understanding of ICI response. DSP revealed that ACTA2 expression in α-SMA^+^ CAFs inversely correlated with survival; patients with low expression achieved a 56% response rate compared to 25% in high-expression groups [122]. Another study highlighted that ICI resistance genes were predominantly expressed in the desmoplastic CAF (dCAF) subtype, where Decorin (DCN) expression negatively correlates with PD-L1; patients with PD-L1^+^ and DCN- exhibited the best response for ICIs [123]. Furthermore, in patients with a high tumor mutational burden (TMB-H, ≥16 mt/Mb), responders showed enrichment of NK cells and cytotoxic T cells in tumor regions, whereas non-responders were dominated by exhausted T cells and M2 macrophages [124]. In ICI-resistant cases, DSP identified significantly increased infiltration of PMN-MDSCs, which suppressed CTL proliferation and overexpressed immunosuppressive molecules such as Arg1 and VISTA, along with phosphorylated S6K and Gli2. Therapeutic intervention with the tyrosine kinase inhibitor cabozantinib depleted MDSCs and restored CTL sensitivity, while rapamycin enhanced CTL-mediated killing by inhibiting the mTOR–GLI axis [125]. Additionally, the co-expression of NKG2A and PD-1 on CD8^+^ T cells defined two patterns, immune checkpoint expression pattern1 (ICEP1, high co-expression of immune checkpoint molecules, ICI-responsive) and ICEP2 (NKG2A-predominant, ICI-resistant). NKG2A binding to HLA-E on tumor cells was a critical driver of immune evasion [126]. In fibrotic tumors including DGC, TGFβ1 enhanced PDGFD-PDGFRβ signaling, promoting CAFs proliferation and the secretion of chemokines (CXCL1, CXCL3) that recruited PMN-MDSCs. The PDGFRα/β inhibitor Regorafenib reduced granulocyte chemotaxis and its combination with anti-PD-1 synergistically increased CD8^+^ T cell and IFNγ^+^ effector T cell infiltration [127]. Further studies in patients resistant to anti-angiogenesis (Regorafenib) and anti-PD-1(Avelumab) combination therapy showed that M2 macrophage infiltration and S100A10 (a macrophage chemoattractant) were overexpressed in tumor compartments, correlating with elevated serum levels of CSF-1 and IL-4 [128] (Figure 2A).

### 5.2. HER2-Targeted Therapy Resistance

In a HER2^+^ GC phase II clinical trial, ST showed that trastuzumab induced NK cell infiltration and Fc receptor gamma III (FCGR3) expression in macrophages to enhance ADCC. TCR signaling of CD8^+^ T cells was higher in PD-L1^+^ regions [129]. Spatial profiling of patient-matched HER2^+^ samples revealed that HER2^+^ GC exhibited higher PD-L1 expression in the tumor stroma than HER2^-^ cases, with no difference in tumor compartments. Trastuzumab resistance was driven by EMT with PD-L1/CCL2 upregulation or endoplasmic reticulum-associated degradation (ERAD) pathway activation, followed by a significant post-resistance rise in CLDN18.2. In contrast, trastuzumab deruxtecan (T-DXd) resistance correlated with HLA transcriptional loss and elevated oxidative phosphorylation. Clinically, the trastuzumab–pembrolizumab synergy is most potent in HER2^+^/PD-L1^+^ patients [130]. Consistent with this, DSP analysis of GC organoids confirmed that HER2 knockdown downregulated PD-L1 via AKT/mTOR inhibition, enhancing CTL-mediated cytotoxicity and organoid death. Although MDSCs compromise these effects, Cabozantinib-mediated MDSC depletion restored sensitivity, providing a robust rationale for combination regimens in GC [131]. During neoadjuvant FLOT plus Pembrolizumab therapy, non-responders exhibited higher proportions of PD-1^+^ CD8^+^ T cells and PD-1^+^FoxP3^+^ Tregs, enriched with LGALS3 and IDO1 [132]. Moreover, PDE5A^+^ CAFs recruited LAG3^+^ Tex via the CXCL12-CXCR4 axis, creating a physical barrier that restricts CTL infiltration; this can be reversed by combining the PDE5A inhibitor Vardenafil with anti-LAG3 antibodies [133]. Additionally, in early GC (EGC), AI-integrated spatial omics identified a precancerous metastatic niche (PMC_P) enriched in stem-like inflammatory pit mucous cells (PMC_2). These cells interacted with macrophages and fibroblasts through the AREG-EGFR and NAMPT-ITGA5 axis to activate JAK-STAT and MAPK pathways, upregulating PD-L1 [134] (Figure 2B).

### 5.3. Resistance in Gastric Cancer Peritoneal Metastasis

Haoxin Peng et al. demonstrated that in GCPM, terminally differentiated MUC1^+^ cancer cells with high EMT potential were spatially proximal to fibroblasts and endothelial cells, a configuration associated with poor prognosis. In the peritoneal microenvironment of chemoresistant patients, C1Q^+^ macrophages exhibited enhanced interactions with fibroblasts and neutrophils via the ANXA1-FPR1 and CCL3-CCR1 pathways, suppressing CTL proliferation and cytotoxicity. Conversely, in immunotherapy-resistant patients, MUC1^+^ cancer cells, C1Q^+^ macrophages and FCER1G^+^ B cells formed a synergistic immunosuppressive network that hindered T cell infiltration and activation. Notably, the bifunctional PD-L1/TGF-βRII inhibitor (SHR-1701) significantly extended progression-free survival in these patients [135]. Another research on resistant GCPM identified a high expression of thrombospondin type 1 domain-containing protein 4 (THSD4) in peritoneal metastases. Primarily localized in dCAFs, THSD4 mediated chemoresistance by activating midkine (MK) and EMT signaling pathways [136]. For chemoresistance, Jang E et al. defined five GC molecular subtypes, identifying the mesenchymal (MSC) and intestinal with stem-like (INT/S) subtypes as having the poorest prognosis. ST revealed partial EMT states and intrinsic TGF-β activation within tumor cells. Importantly, TGF-β inhibition could reverse these mesenchymal/stem-like phenotypes, effectively restoring chemosensitivity [137] (Figure 2C).

### 5.4. The Role of TLSs and CSCs

The spatial distribution of TLSs is a critical determinant of immunotherapy efficacy and prognosis in GC [138,139]. Yanchun Wang et al. categorized TLSs into intra-tumoral (iTLS), peritumoral (pTLS), and TLS-deficient (dTLS) groups. Integrated scRNA-seq and ST analysis revealed that patients possessing iTLS exhibited the most robust ICIs response and longest survival. These iTLS regions were enriched with CXCL13^+^ T cells, germinal center B cells, LAMP3^+^ activated dendritic cells and SELP^+^ACKR1^+^ endothelial-venule (HEV) cells [140]. Similarly, another study showed that ICI responders possessed higher TLS density/maturity and increased CXCL13^+^ CD160^+^ CD8^+^ T cell infiltration compared to non-responders. B cell recruitment for TLSs formation was driven by the CXCL13-CXCR5 axis. Furthermore, Vitamin B6 could enhance PD-1 inhibitor efficacy by inhibiting MDM2-mediated ubiquitination of HIF-1α, thereby upregulating CXCL13 secretion [141]. Cancer stem cells (CSCs) remain a primary driver of therapeutic resistance. Guangyu Zhang’s team demonstrated that GC CSCs originated from SPEM cells via transdifferentiation of mature gastric chief cells, with their abundance correlating with poor prognosis. Spatially, CSCs co-localized with iCAFs and immunosuppressive cells, evading immune surveillance through checkpoint pathways involving CXCL13^+^ T cells and CCL18^+^ M2 macrophages. As a core component of the CSC niche, iCAFs upregulated SOX9 in CSCs via the AREG-ERBB2 axis, promoting stemness and chemoresistance—a process effectively inhibited by the ERBB2 inhibitor Lapatinib [142] (Figure 3D).

Although spatial omics has advanced the understanding of therapeutic resistance in GC, several gaps remain. The same problem, most studies rely on spatial transcriptomic data, lacking deep integration with spatial proteomics, metabolomics, epigenomics and translatomics. Furthermore, current research focuses predominantly on immunotherapy and HER2-targeted therapy. More extensive studies are needed to elucidate the spatial mechanisms of resistance to other targeted agents and anti-angiogenic treatments, as well as to distinguish between different pathological subtypes, such as diffuse and intestinal types.

## 6. Future and Perspectives

With its distinct advantages in spatial localization and molecular profiling, spatial multi-omics has achieved significant breakthroughs in optimizing molecular subtyping, screening novel biomarkers, characterizing heterogeneity and elucidating therapeutic resistance in GC. These advancements provide a transformative perspective for precision oncology. However, the field is still in its early stages, and several bottlenecks hinder its application in GC. Future efforts must prioritize technological innovation, the expansion of research dimensions and the deepening of clinical translation.

Current spatial multi-omics studies largely rely on multidimensional analysis across serial sections. Even with consecutive sections, maintaining perfect histological consistency is challenging, leading to potential data bias and compromised accuracy. Recently, Rong Fan’s team developed “spatial multi-omics co-detection” technologies enabling the simultaneous acquisition of transcriptomic and proteomic data from a single tissue section [53]. This approach effectively mitigates errors arising from sectional variation. Nevertheless, several technical limitations persist. First, the throughput remains limited, currently covering only hundreds of genes and dozens of proteins, which falls short of the requirements for global, panoramic analysis of the whole transcriptome or proteome. Second, the stringent requirement for high-quality fresh frozen tissue renders these technologies incompatible with standard FFPE samples, thereby restricting retrospective clinical research. Despite these hurdles, as technologies continue to evolve, multidimensional spatial multi-omics detection on single tissue sections is expected to become increasingly refined and robust.

As a digestive malignancy, GC development is intimately linked to interactions with microorganisms such as *Helicobacter pylori*. However, current spatial omics research has yet to fully address the spatial distribution of the intra-tumoral microbiota and its interplay with malignant cells and the immune microenvironment. Studies in other cancer types utilizing DSP have successfully elucidated how intra-tumoral microbes modulate progression, offering a roadmap for GC research [143]. Future studies should integrate spatial transcriptomics and metabolomics with microbial sequencing to precisely map microbial niches. This will clarify their spatial associations with metabolic reprogramming and immunosuppressive niches, ultimately uncovering the microbial drivers of gastric oncogenesis. Furthermore, current GC research predominantly focuses on integrating spatial transcriptomics with scRNA-seq, leaving spatial genomics, epigenomics and translatomics underutilized. This fragmentation hinders a comprehensive understanding of the regulatory continuum from genetic variation to phenotypic expression. Future efforts must emphasize the synergistic application of diverse spatial technologies—integrating genomic, transcriptomic, proteomic and metabolic data—to construct a “spatial molecular regulatory network” that captures multi-layered cooperative mechanisms.

Regarding clinical translation, most novel biomarkers and therapeutic targets identified via spatial omics remain in the discovery phase. They lack large-scale, multi-center clinical validation, standardized detection protocols and established interpretive criteria. Notably, several typical pilot clinical application cases of spatial multi-omics in GC have emerged, laying the foundation for broader clinical translation: (1) Early diagnosis of precancerous lesions: spatial transcriptomics has been applied to map molecular abnormalities in gastric intestinal metaplasia, identifying region-specific upregulation of GPRC5A in high-risk lesions—this marker exhibits 89% specificity for distinguishing precancerous lesions from normal mucosa, providing a high-precision tool for early GC screening [16]. (2) Individualized treatment guidance for advanced GC: spatial metabolomics has guided therapeutic selection in HER2+ advanced GC, where patients with high metabolic heterogeneity (Simpson Index > 0.7) showed a 67% response rate to trastuzumab monotherapy, while those with low heterogeneity (Simpson Index < 0.5) achieved a 58% response rate with trastuzumab combined with pertuzumab, demonstrating the value of spatial metabolic profiling in personalized regimen design [67]. To facilitate clinical implementation, it is essential to establish standardized workflows for sample processing and data analysis while developing rapid, cost-effective clinical platforms. In terms of standardization progress, recent advancements include optimized FFPE sample processing protocols for spatial transcriptomics (e.g., Patho-DBiT technology) that maintain RNA integrity and spatial resolution with inter-laboratory reproducibility > 90%, and the development of standardized data analysis pipelines (e.g., iStar) for cell type deconvolution and spatial signature extraction, reducing technical variability [24]. Simultaneously, prospective clinical trials are needed to validate the predictive value of spatial molecular signatures for therapeutic response and prognosis. Moreover, tailored research designs for pathological subtypes (e.g., diffuse and intestinal types) are necessary to provide a precise theoretical foundation for individualized treatment. Additionally, it is crucial for gastric cancer to be managed at high-volume medical centers, as the specialized experience and rigorous omics-related research accumulated there are not easily replicable across all institutions.

In conclusion, spatial multi-omics holds immense promise for GC research. By overcoming existing bottlenecks through technological innovation, expanding research dimensions to uncover novel mechanisms and bridging the gap to clinical application, the field is poised to transition from “panoramic analysis” to “precision intervention”, offering new hope for improving outcomes in patients with gastric cancer.

## Figures and Tables

**Table 1:** Summary of Spatial Multi-omics Technologies in Cancer Research.

Technology Layer	Detection Principle	Representative Platforms	Spatial Resolution	Key Strengths and Limitations	Application in GC
Transcriptomics	NGS-based: spatial barcoding and mRNA capture	10× Visium (HD)	500 nm–55 μm	Pros: Whole-transcriptome coverage.	Molecular classification, biomarker discovery, decoding heterogeneity, drug resistance mechanism analysis
		Slide-seqV2	10 μm	Cons: Diffusion effects; variable sensitivity.	
		Stereo-seq	0.5 μm		
		DBiT-seq	10 μm		
	Image-based: cyclic ISH and combinatorial coding	MERFISH	Subcellular (<1 μm)	Pros: High detection efficiency; single-molecule resolution.	
		seqFISH+		Cons: Limited gene plex; complex imaging.	
Proteomics	MS-based; metal-tagged antibodies and laser/ion ablation	IMC	1 μm (subcellular)	Pros: High multiplexing (40+ proteins); no autofluorescence.	Molecular classification, decoding heterogeneity
		MIBI-TOF		Cons: Destructive to tissue; low throughput.	
	Antibody-based; DNA-barcoding/photocleavable indexing	PhenoCycler (CODEX)	0.5 μm (CODEX)	Pros: High-plex protein imaging; FFPE compatible.	
		GeoMx DSP	Region-based (DSP)	Cons: Long cycle times (CODEX); not true single-cell (DSP).	
Translatomics	Probes for ribosome-bound mRNA + in situ sequencing	RIBOmap	Subcellular	Pros: Direct mapping of protein synthesis activity.	
				Cons: Limited gene throughput; experimental complexity.	
Genomics	ISH-based or barcoded sequencing	DNA MERFISH	Subcellular to 10 μm	Pros: Resolves CNVs and clonal evolution spatially.	
		Slide-DNA-seq		Cons: Technical difficulty; low genomic coverage.	
Epigenomics	Tn5 transposase-mediated in situ barcoding	Spatial ATAC-seq	20–50 μm	Pros: Maps chromatin accessibility and histone marks.	
		Spatial CUT&Tag		Cons: High background noise; restricted to fresh tissue.	
Metabolomics	Mass spectrometry imaging (MSI)	MALDI-MSI	<1 μm (SIMS) to 200 μm (DESI)	Pros: Unlabeled detection of lipids/metabolites.	Molecular classification, decoding heterogeneity
		DESI-MSI		Cons: Vacuum requirements (MALDI); low resolution (DESI).	
		SIMS			
Spatial Multi-omics	ST + ATAC	Spatial ATAC-RNA-seq	High-resolution (10–20 μm)	Pros: Co-profiles chromatin accessibility and gene expression; uncovers regulatory relationships (e.g., enhancer–promoter interactions).	
				Cons: Complex workflow; restricted to fresh tissue.	
	ST + ATAC	MISAR-seq	High-resolution (10–20 μm)	Pros: Integrates spatial barcoding with transposase-mediated epigenetic labeling and mRNA capture; identifies key transcription factors and gene regulatory networks.	
				Cons: Microfluidic-based operation requires specialized equipment.	
	ST + DNA Methylation	Spatial DNA methylation and transcription detection	High-resolution (10–20 μm)	Pros: Enables simultaneous spatial detection of DNA methylation and gene expression using spatial barcoding technology.	
				Cons: Technical details and broad applicability remain to be fully validated.	
	ST + Histone Modification	Spatial CUT&Tag-RNA-seq	High-resolution (10–20 μm)	Pros: Simultaneously profiles histone modifications and RNA expression; provides high-resolution nucleosome positioning.	
				Cons: High background noise; dependent on specific antibodies.	
	ST + Proteomic (ADTs)	Spatial-CITE-seq	10 μm	Pros: Retains whole-transcriptome coverage	
				Cons: Lacks true single-cell resolution; ADT panel size is limited.	
	ST + Proteomic (ADTs)	Stereo-CITE-seq	0.5 μm (subcellular)	Pros: Leverages DNA nanoball arrays; achieves precise spatial correlation of transcriptomics and proteomics	
				Cons: Compatible only with fresh-frozen tissues; high experimental cost.	
	ST + Proteomic	GeoMx DSP	10–600 μm	Pros: Compatible with FFPE samples; supports flexible region-specific ROI selection; enables targeted co-detection of RNA and proteins.	Molecular classification, decoding heterogeneity, drug resistance mechanism analysis
			Region-based (ROI-selective)	Cons: Not true single-cell resolution; relatively lower throughput.	
	ST + Proteomic (ADTs)	SPOTS	55 μm	Pros: Uses Visium-like oligo(dT)-incubated arrays; simple workflow; compatible with fresh-frozen and FFPE samples.	
				Cons: Limited multiplexing capability compared to other platforms.	
	ST + Proteomic	SM-Omics	100 μm	Pros: Visium-like array-based capture; high-throughput; automated operation for large-scale transcriptome–proteome co-profiling.	
				Cons: Requires specialized automation equipment; data analysis pipeline is complex.	
	ST + Proteomic	STARmap PLUS	0.2–0.3 μm (subcellular)	Pros: Combines antibody staining and chemical labeling; ultra-high spatial resolution.	
				Cons: Complex staining and labeling procedures; limited to specific tissue types.	
	Targeted ST + Proteomic	MOSAICA	3 μm	Pros: Uses primary hybridization probes and double-ended secondary probes; incorporates lifetime imaging and combinatorial encoding with error-correction cycles; suitable for complex tissue samples.	
				Cons: Long experimental cycle; requires advanced imaging and data decoding systems.	

**Table 2:** Novel Biomarkers in Gastric Cancer Identified via Spatial Omics Technologies.

Biomarker	Full Name	Source Cell/Localization	Functional Role (Prognostic/Therapeutic)	Ref
Metabolism and Proliferation-Related Markers				
GPRC5A	G Protein-Coupled Receptor Class C Group 5 Member A	Tumor core cells	Diagnostic/Prognostic: Early diagnostic marker linked to enhanced glycolysis.	[75]
RRM2	Ribonucleotide Reductase Regulatory Subunit M2	Hyper-proliferative cell clusters	Therapeutic: Regulates ferroptosis; inhibitors (e.g., Osalmid) show anti-tumor potential.	[76]
NDUFAB1	NADH:Ubiquinone Oxidoreductase Subunit AB1	Tumor–stroma interface	Prognostic/Therapeutic: Driver of progression; regulates cell cycle genes and stemness.	[77]
NFIX	Nuclear Factor I X	Cancer1 subpopulation (CSC)	Therapeutic: Key molecule regulating cancer stem cell-like properties.	[78]
IQGAP3	IQ Motif Containing GTPase Activating Protein 3	Gastric proliferative stem cells	Prognostic: Hub for KRAS/TGF-beta maintains proliferative and slow-cycling niches.	[79]
Immune Microenvironment-Based Markers				
CCL28	C-C Motif Chemokine Ligand 28	GC cells/IgA+ plasma cells	Therapeutic: Synergizes with anti-PD-L1 to enhance anti-tumor efficacy.	[80]
SPP1	Secreted Phosphoprotein 1	M2 macrophages (deep tissue)	Prognostic: Activates pro-tumorigenic pathways via SPP1/CD44 axis; poor prognosis.	[81]
CCL2	C-C Motif Chemokine Ligand 2	CCL2+ CAFs	Therapeutic: Recruits myeloid cells to create immunosuppression; neutralizing targets.	[82,83,84]
CXCR4	C-X-C Motif Chemokine Receptor 4	Tregs (oxidative stress zones)	Therapeutic: Associated with immunotherapy resistance.	[85]
ALKBH1	AlkB Homolog 1, Histone Demethylase	Spatially enriched tumor regions	Prognostic/Therapeutic: Independent prognostic marker and immunotherapy target.	[86]
TCTP	Translationally Controlled Tumor Protein	Tumor cells	Prognostic: Limits T cell infiltration; poor prognosis for chemo/immunotherapy.	[87]
Markers in Specialized Pathological and Environmental Contexts				
TMEM176B	Transmembrane Protein 176B	Young GC (GCY) tumor core	Therapeutic: Potential target regulating proliferation and apoptosis in GCY.	[88]
AFB1	Aflatoxin B1	Gastric cancer cells	Therapeutic: Drive GC cells to immune escape via the MAPK3–FOXM1–Cyclin E axis.	[89]
CD44	Cluster of Differentiation 44	Neutrophils (CD44_NEU)	Therapeutic: Predicts low response to immune checkpoint inhibitors (ICIs).	[90]
MZB1	Marginal Zone B And B1 Cell-Specific Protein	Mature TLS/plasma cells	Prognostic/Therapeutic: High MPS score predicts better neoadjuvant PD-1 response.	[91]
SARIFA	Stroma AReactive Invasion Front Areas	Tumor–adipocyte contact areas	Prognostic: High FABP4 in macrophages at the front indicates poor overall survival.	[92]

## Data Availability

No new data were created or analyzed in this study.

## Abbreviations

The following abbreviations are used in this manuscript:

GC	Gastric cancer
TME	Tumor microenvironment
ST	Spatial transcriptomics
NGS	Next-Generation Sequencing
FFPE	Formalin-fixed paraffin-embedded
DBiT-seq	Deterministic Barcoding in Tissue for spatial omics sequencing
ISH	In situ hybridization
IMC	Imaging Mass Cytometry
MIBI-TOF	Multiplexed Ion Beam Imaging
DSP	Digital Spatial Profiling
ROI	Region of Interest
CNV	Copy number variation
MSI	Mass spectrometry imaging
MALDI-MSI	Matrix-assisted laser desorption/ionization mass spectrometry imaging
DESI-MSI	Desorption electrospray ionization–mass spectrometry imaging
SIMS	Secondary ion mass spectrometry
TCGA	The Cancer Genome Atlas
ACRG	Asian Cancer Research Group
EBV	Epstein–Barr virus
MSI	Microsatellite Instability
CIN	Chromosomal Instability
GS	Genomically Stable
ITH	Intra-tumoral heterogeneity
MSC	Metabolic signature cluster
MP	Meta-program
PCC	Poorly cohesive carcinoma
CSC	Cancer stem cell
CAF	Cancer-associated fibroblast
DGC	Diffuse Gastric Cancer
SRC	Signet ring cell
PDC	Poorly differentiated cell
GSRC	Gastric signet ring cell carcinoma
ICI	Immune checkpoint inhibitor
CPS	Combined positive score
TMB-H	High tumor mutational burden
PMN-MDSC	Polymorphonuclear myeloid-derived suppressor cell
CTL	Cytotoxic T lymphocyte
ICEP	Immune checkpoint expression pattern
ERAD	Endoplasmic reticulum-associated degradation
T-DXd	Trastuzumab deruxtecan
PMC_P	Precancerous metastatic niche
GCPM	Gastric cancer peritoneal metastasis
THSD4	Thrombospondin type 1 domain-containing protein 4
INT/S	Intestinal with stem-like features
TLS	Tertiary lymphoid structure
iTLS	Intra-tumoral tertiary lymphoid structure
pTLS	Peritumoral tertiary lymphoid structure
HEV	High endothelial venule
GCY	Young gastric cancer
TCR	Tumor core region
CAT	Cancer-adjacent tissues
SARIFA	Stroma AReactive Invasion Front Areas
